# Pathological Evolution of Fibrosis in IgG4‐Related Ophthalmic Disease: A Retrospective Study of 100 Cases

**DOI:** 10.1111/pin.70193

**Published:** 2026-10-08

**Authors:** Natsuki Taniguchi, Akira Satou, Yasuharu Sato, Taishi Takahara, Yasuhiro Takahashi, Toshinobu Kubota, Akari Iwakoshi, Toyonori Tsuzuki

**Affiliations:** ^1^ Department of Surgical Pathology Aichi Medical University Hospital Nagakute Aichi Japan; ^2^ Department of Pathology and Laboratory Medicine Nagoya University Hospital Nagoya Aichi Japan; ^3^ Department of Molecular Hematopathology Okayama University Graduate School of Health Sciences Okayama Okayama Japan; ^4^ Department of Oculoplastic, Orbital and Lacrimal Surgery Aichi Medical University Hospital Nagakute Aichi Japan; ^5^ Department of Ophthalmology National Hospital Organization Nagoya Medical Center Nagoya Aichi Japan; ^6^ Department of Pathology National Hospital Organization Nagoya Medical Center Nagoya Aichi Japan

**Keywords:** CD163‐positive macrophages, fibrosis, IgG4‐related ophthalmic disease, lacrimal gland, *TGFB1*

## Abstract

IgG4‐related ophthalmic disease (IgG4‐ROD) is a common manifestation of IgG4‐related disease, but its clinicopathologic features and the mechanisms underlying fibrosis remain incompletely understood. We retrospectively analyzed 100 cases of IgG4‐ROD and investigated fibrosis using immunohistochemistry for IgG4, FOXP3, CD68, and CD163, together with RNA in situ hybridization for *TGFB1*. IgG4‐ROD was histologically categorized into four patterns (Patterns 1–4) reflecting progressive fibrosis, demonstrating a spectrum from lymphoplasmacytic inflammation to marked fibrosis. Fibrosis was absent or minimal in 51% of cases, indicating that fibrosis is not essential for diagnosis, whereas obliterative phlebitis was rare. Compared with non‐fibrotic cases, fibrotic cases showed significantly increased numbers of CD68‐positive and CD163‐positive macrophages (*p* < 0.001), predominantly localized at the interface between lymphoid and fibrotic areas. In contrast, fibrosis was not associated with IgG4‐positive plasma cells, FOXP3‐positive regulatory T cells, or EBV status. *TGFB1* expression was significantly higher in lymphocyte‐rich than fibrotic areas (*p* < 0.001), suggesting a closer association with lymphocyte‐rich inflammation than established fibrosis. These findings demonstrate progressive histologic changes from Pattern 1 to Pattern 4 and suggest that accumulation of CD163‐positive macrophages is associated with fibrosis in IgG4‐ROD.

## Graphical abstract

IgG4‐related ophthalmic disease comprises four histologic patterns representing progressive fibrosis and sclerosis. Fibrosis is associated with CD163‐positive M2 macrophages and increased TGFB1 expression in lymphocyte‐rich areas, suggesting roles for these components in disease progression.

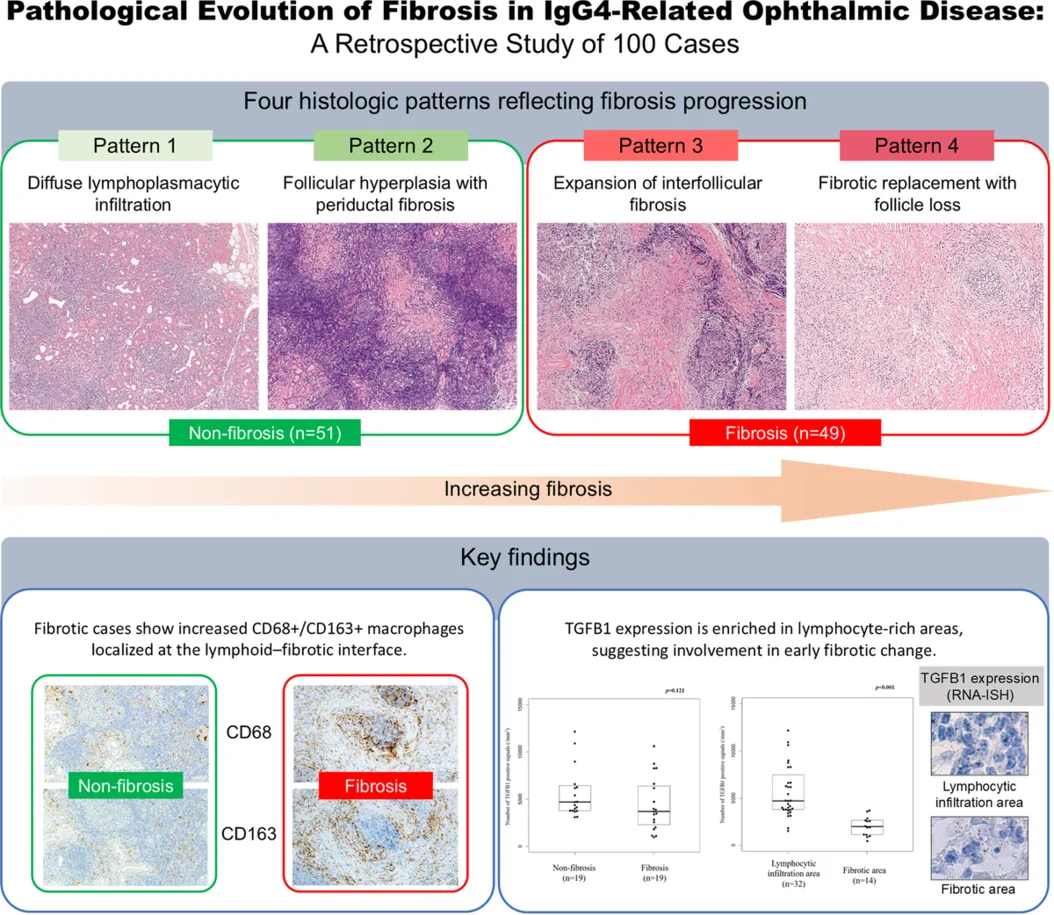

## 1 Introduction

IgG4‐related disease (IgG4‐RD) is characterized by prominent IgG4‐positive lymphoplasmacytic infiltration and fibrosclerosis into multiple organs—such as the pancreas, bile duct, lung, kidney, aorta, retroperitoneum, major salivary glands, and lacrimal glands [1, 2, 3]. The ocular orbit is a frequent site of involvement, with the majority of cases presenting with lacrimal gland lesions [4, 5, 6]. Patients with IgG4‐related dacryoadenitis commonly also exhibit lesions in the major salivary glands, a condition formerly known as Mikulicz's disease [7, 8]. Japanese studies show that lymphoproliferative disorders (LPDs) account for approximately 38%–49% of orbital tumors [9, 10]. Among these orbital LPDs, around 25% are diagnosed as IgG4‐related ophthalmic disease (IgG4‐ROD) [11]. Although the orbit is a major site affected by IgG4‐RD, comprehensive clinicopathologic analyses of IgG4‐ROD remain limited [4, 5, 12].

In cases of IgG4‐RD, fibrosis is a major histologic finding. TGF‐β is a central mediator of organ fibrosis, promoting fibroblast activation and extracellular matrix deposition across diverse tissues [13, 14], and is regarded as a key contributor to fibrotic processes in IgG4‐RD. Chinju et al. have reported that, in IgG4‐RD, CD163‐positive M2 macrophages contribute to fibrosis by enhancing the production of fibrotic cytokines, including TGF‐β, through activation of the TLR‐7/IRAK4/NF‐κB signaling pathway [15]. Zen et al. propose that IgG4‐RD is characterized by the overactivation of T helper 2 (Th2) cells and regulatory T cells (Tregs) [16], and that Tregs may contribute to local production of IL‐10 and TGF‐β, the latter of which plays a direct role in driving fibrosis in IgG4‐RD. However, few studies have visually identified Tregs, TGF‐β, and M2 macrophages in tissue specimens from patients with IgG4‐RD, and analyzed their association with fibrosis.

To better characterize IgG4‐ROD, in this study, we performed a clinicopathological analysis of 100 cases. Tissue specimens were subjected to immunohistochemical analyses and RNA in situ hybridization for *TGFB1*, to visually investigate the mechanisms of fibrosis in IgG4‐ROD. To our knowledge, this represents the largest cohort study to date focusing on detailed histopathological evaluation of IgG4‐ROD.

## 2 Materials and Methods

### 2.1 Patient Samples

We retrospectively retrieved 106 cases evaluated between 2009 and 2022 at two hospitals: Aichi Medical University Hospital and National Hospital Organization Nagoya Medical Center, including cases diagnosed as IgG4‐ROD according to the diagnostic criteria available at the time and cases in which IgG4‐ROD had been considered in the clinical and/or histopathological differential diagnosis (Supporting Information Figure S1). The 106 cases were independently reviewed by two pathologists (authors N.T. and A.S.) in accordance with the 2023 revised diagnostic criteria for IgG4‐ROD (Supporting Information Table S1) [17]. Cases histologically suspicious for MALT lymphoma were evaluated for light‐chain restriction by in situ hybridization. Five cases showing light‐chain restriction were reclassified as MALT lymphoma and excluded from the analysis. One additional case was excluded because no tissue block was available for immunohistochemical analysis. Among the remaining 100 cases, 97 fulfilled the criteria for a definite diagnosis and three for a probable diagnosis. Clinical, laboratory, and follow‐up data were obtained from the patient medical records at each institution. The lesion sites were typically examined via biopsy or radiographic evaluation, for example, computed tomography or positron emission tomography. Histological specimens from extra‐ophthalmic lesions were generally unavailable for review. The study protocol was approved by the institutional review boards of Aichi Medical University Hospital (approval no. 2021‐066) and National Hospital Organization Nagoya Medical Center (approval no. 2022‐012). Informed consent was obtained using an opt‐out approach.

### 2.2 Histological and Immunohistochemical Staining

Tissue samples were fixed in 10% formalin, embedded in paraffin, and cut into 4‐μm‐thick sections, which were stained with hematoxylin and eosin (H&E). Immunohistochemical staining of formalin‐fixed paraffin‐embedded (FFPE) section was conducted using automated immunostainers (Bond‐III; Leica and Benchmark/Ultra; Roche). Heat‐induced antigen retrieval was performed for each antibody, followed by application of the antibodies. The following monoclonal antibodies were used: CD138 (MI15; Dako, Santa Fe, CA), IgG (A57H; Nichirei, Tokyo, Japan), IgG4 (HP6025; Invitrogen, Carlsbad, CA), CD68 (KP1; Dako, Santa Fe, CA), CD163 (10D6; DAKO, Santa Fe, CA), and FoxP3 (236 A/E7; Abcam, Cambridge, UK). The numbers of CD138‐, IgG‐, and IgG4‐positive cells were counted in a representative high‐power field exhibiting the highest density of positive cells. Three representative high‐power fields were selected to evaluate the expressions of CD68, CD163, and FoxP3. Positive cells were counted in each field, and the average number of positive cells was determined.

### 2.3 In Situ Hybridization Study

In situ hybridization (ISH) was performed to examine the presence of Epstein–Barr virus (EBV)‐specific small RNAs. Briefly, to FFPE sections, we applied EBV‐encoded small nuclear early region (EBER) oligonucleotides, as previously described [18].

RNA ISH of *TGFB1* was conducted using a specific probe targeting *TGFB1* (Advanced Cell Diagnostics, Newark, CA, USA), as previously described [19]. Procedures were conducted using the RNAscope 2.5 HD Reagent Kit BROWN according to the manufacturer's instructions. Briefly, FFPE sections underwent deparaffinization, pretreatment, probe hybridization, signal amplification, and DAB detection, followed by hematoxylin counterstaining. The slides were evaluated under light microscopy, digital images were acquired using an Olympus BX53 light microscope (Olympus, Tokyo, Japan), and these images were analyzed using ImageJ software (National Institutes of Health, Bethesda, MD, USA).

Brown punctate dots in the cytoplasm were interpreted as positive signals representing *TGFB1* mRNA expression, and were quantified per unit area (mm^2^). For each case, the representative *TGFB1* expression value was defined as the signal count measured in the area exhibiting the highest *TGFB1* expression within the tissue section. To assess the intra‐case heterogeneity of *TGFB1* expression, tissue sections were histologically assessed and classified into two distinct regions: lymphocytic infiltration areas and fibrotic areas. A region was defined as lymphocytic or fibrotic when over 80% of the field comprised dense lymphocytic infiltration or dense fibrotic stroma, respectively. When both regions were present within the same specimen, *TGFB1* expression was quantified separately in each region. In cases where only one region was identifiable, analysis was performed in the area showing the highest *TGFB1* expression within that compartment.

### 2.4 Statistical Analysis

Correlations between the two groups were analyzed using the *χ* ^2^ test, Fisher's exact test, and Kruskal–Wallis test, as appropriate. Results with *p* < 0.05 were considered significant. All statistical analyses were performed using STATA software, version 12 (STATA Corporation, College Station, TX, USA).

## 3 Results

### 3.1 Patient Characteristics

Table 1 summarizes the clinical characteristics of 100 patients with IgG4‐ROD. The patients included 51 males and 49 females, with a median age of 58.5 years (range, 28–88 years). According to the diagnostic criteria (Supporting Information Table S1), 97 cases were classified as definite IgG4‐ROD, and three as probable IgG4‐ROD because they exhibited serum IgG4 levels below the reference range. Whole‐body imaging was performed in 93 of the 100 patients, which revealed extra‐ophthalmic lesions in 41 of these cases, including in the salivary gland (*n* = 31), lymph node (*n* = 11), pancreas (*n* = 6), retroperitoneum (*n* = 5), kidney (*n* = 3), pituitary gland (*n* = 1), and lung (*n* = 1). Laboratory data from the time of presentation revealed elevated soluble interleukin‐2 receptor (sIL‐2R) levels in 53 of 85 patients (62%). Follow‐up and treatment information was available for 94 patients, all of whom received glucocorticoid treatment. Of these, 28 patients were able to discontinue glucocorticoids at least once during follow‐up. Two patients died due to causes not associated with IgG4‐RD.

**TABLE 1:** Clinicopathological characteristics of patients with IgG4‐related ophthalmic disease (IgG4‐ROD).

	IgG4‐ROD (*n* = 100)
Demographics	
Sex, male/female	51/49
Age, years	58.5 (28–88)
Clinical findings	
Involvement of other sites	44% (41/93)
Salivary gland	33% (31/93)
Lymph node	12% (11/93)
Pancreas	6.5% (6/93)
Retroperitoneum	5.3% (5/93)
Kidney	3.2% (3/93)
Pituitary gland	1.1% (1/93)
Lung	1.1% (1/93)
Serum IgG4, mg/dL	459 (65.5–3700)
sIL‐2R > normal	97% (97/100)
sIL‐2R, U/mL	596 (121–2481)
sIL‐2R > normal	62% (53/85)
Pathological findings	
Storiform fibrosis	26% (26/100)
Obliterative phlebitis	1% (1/100)
Eosinophil infiltration	35% (35/100)
Germinal center hyperplasia	69% (69/100)
PTGC‐like change	34% (34/100)

*Note:* Data are presented as percentage (proportion) or median (range). The reference cutoff for serum IgG4 was 135 mg/dL. Reference ranges for sIL‐2R were institution‐specific.

Abbreviations: IgG4, immunoglobulin G4; PTGC, progressive transformation of germinal centers; sIL‐2R, soluble interleukin‐2 receptor.

### 3.2 Histological Findings

Histological samples were obtained from the lacrimal gland in 85 cases and from extra‐lacrimal sites in the remaining 15, including 13 from the extraocular muscles. Histologically, all cases exhibited infiltration of lymphocytes and plasma cells. The IgG4‐ROD cases were classified into four patterns (Patterns 1–4). Pattern 1 (*n* = 25) was characterized by abundant infiltration of lymphocytes and plasma cells into the lacrimal glands and the surrounding soft tissue (Figure 1A). Pattern 2 (*n* = 26) exhibited the histological features of Pattern 1, with the additional finding of hyperplastic lymphoid follicles with germinal centers (GCs) and fibrosis confined to the periductal area (Figure 1B). Pattern 3 (*n* = 42) was further characterized by more extensive fibrosis involving the interfollicular area (Figure 1C). Finally, Pattern 4 (*n* = 7) displayed a marked reduction of lymphoid follicles, which was predominantly replaced by fibrosis or scar tissue (Figure 1D).

**FIGURE 1:**
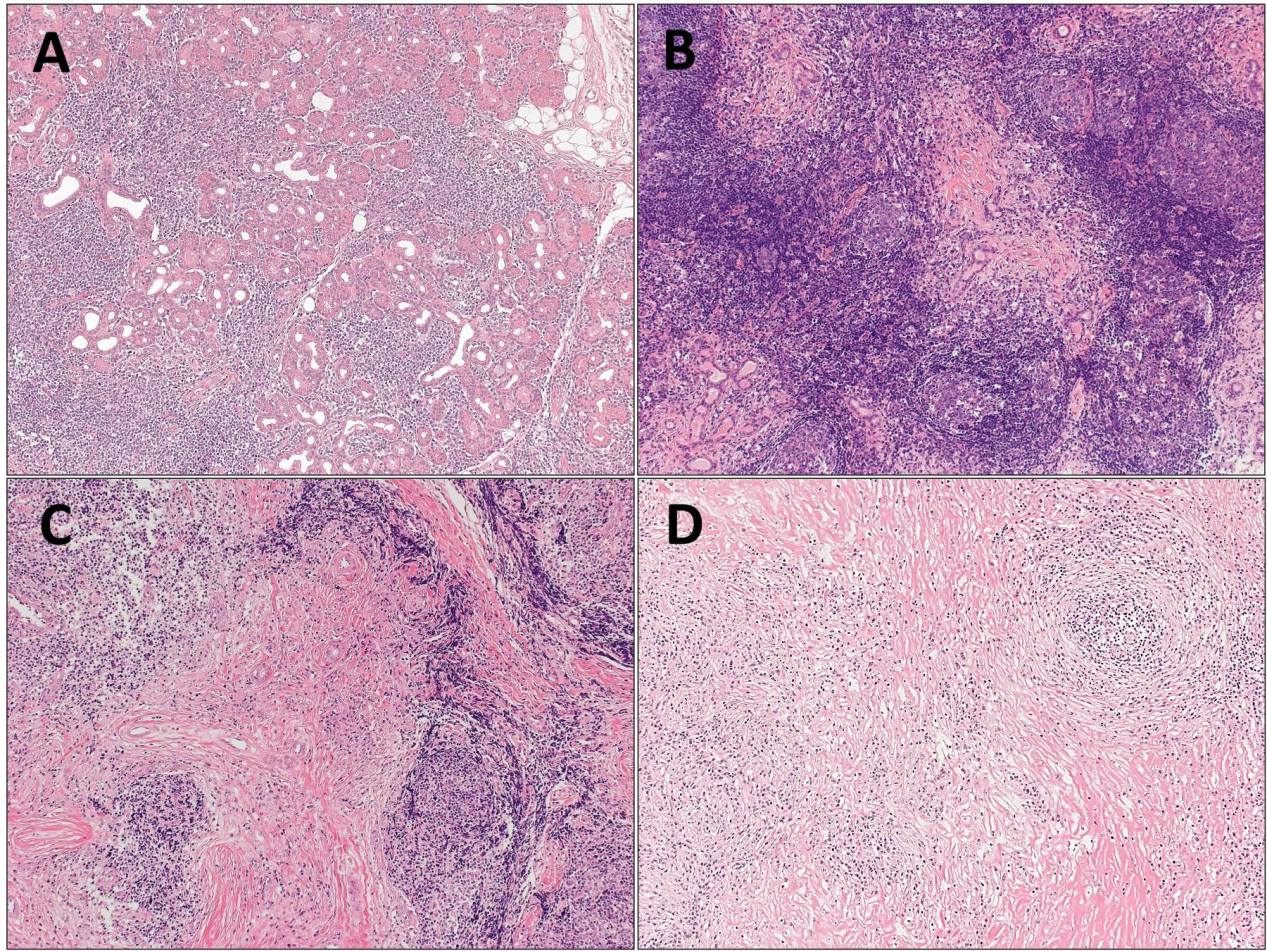
Four histologic patterns of IgG4‐related ophthalmic disease. (A) Pattern 1 was characterized by abundant infiltration of lymphocytes and plasma cells into the lacrimal glands and the surrounding soft tissue (H&E, 40×). (B) Pattern 2 additionally exhibited hyperplastic lymphoid follicles, with germinal centers and fibrosis confined to the periductal area (H&E, 100×). (C) Pattern 3 was characterized by more extensive fibrosis involving the interfollicular area (H&E, 100×). (D) Pattern 4 showed a marked reduction in lymphoid follicles, with extensive replacement by fibrosis or scar tissue (H&E, 100×).

Storiform fibrosis was identified in 26 cases (Supporting Information Figure S2A), and was occasionally observed in Pattern 2, where fibrosis was confined to the periductal area. Only one case exhibited obliterative phlebitis (Supporting Information Figure S2B). Eosinophil infiltration was present in 31 cases (Supporting Information Figure S2C). Lymphoid follicle formation was observed in 78 cases—among which GC hyperplasia was noted in 69 cases, and progressive transformation of germinal centers (PTGC)‐like changes in 34 (Supporting Information Figure S2D).

### 3.3 Immunohistochemical Findings and Distribution of IgG4‐Positive Cells

Among the 100 cases, the number of IgG4‐positive cells ranged from 43 to 602 (median: 224). The ratio of IgG4‐positive to total IgG‐positive cells ranged from 0.30 to 1.00, with a median value of 0.86. Among the 78 cases exhibiting GCs, 42 showed IgG4‐positive cells in both the GCs and interfollicular areas (Figure 2A), while IgG4‐positive cells were confined to interfollicular areas in the other 36 cases (Figure 2B).

**FIGURE 2:**
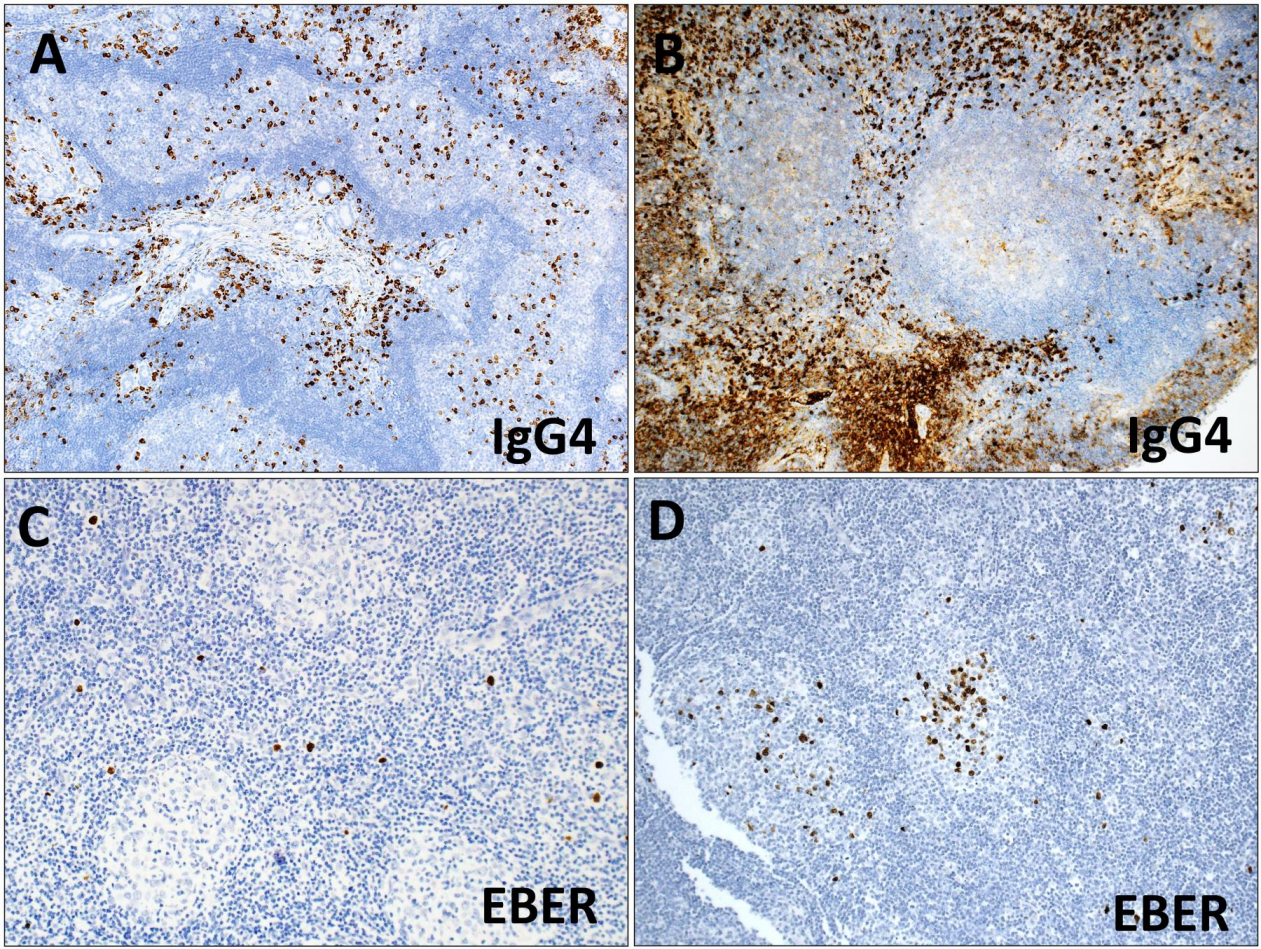
Distribution of IgG4‐positive and EBER‐positive cells. (A) Among the 78 cases with germinal centers (GCs), 42 exhibited IgG4‐positive cells in both the GCs and interfollicular areas (IgG4, 100×). (B) In the remaining 36 cases with GCs, IgG4‐positive cells were confined to interfollicular areas (IgG4, 100×). (C) In most cases, EBER‐positive cells were distributed in the interfollicular or extrafollicular areas (EBER, 200×). (D) Two cases exhibited clusters of EBER‐positive cells within the GCs, with additional positive cells in the interfollicular areas (EBER, 200×).

The number of FOXP3‐positive cells ranged from 7 to 356 (median: 78). FOXP3‐positive cells were observed throughout the sampled tissue, without exhibiting any specific distribution. EBER‐positive lymphocytes were detected in 43 cases, among which 18 exhibited ≥ 5 EBV‐positive cells in at least one high‐power field. In most cases, EBER‐positive cells were distributed in the interfollicular or extrafollicular areas (Figure 2C). In two cases, clusters of EBER‐positive cells were observed within the GCs, with additional positive cells present in the interfollicular areas (Figure 2D).

The number of CD68‐positive cells ranged from 25.3 to 501 (median: 114), and the number of CD163‐positive cells ranged from 22.7 to 412 (median: 145). These CD68‐positive and CD163‐positive cells—considered to be macrophages—exhibited a characteristic distribution, predominantly localizing at the interface between fibrotic areas and lymphoid follicles (Figure 3A–D).

**FIGURE 3:**
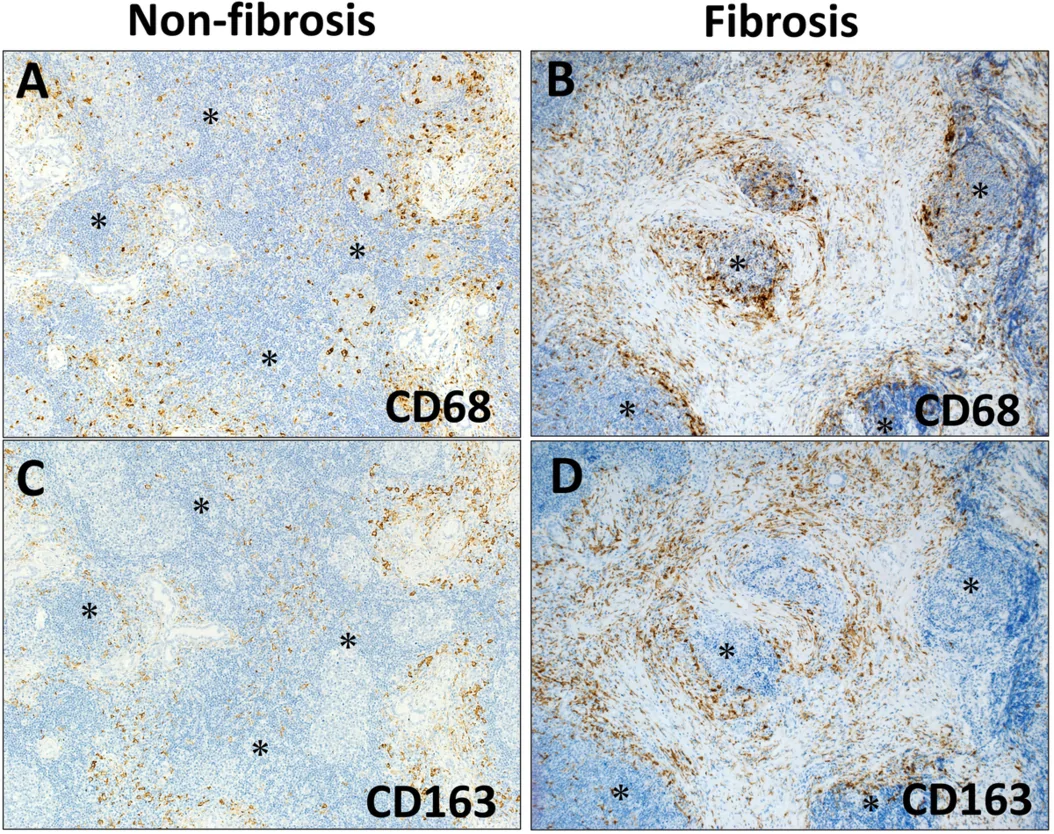
Distribution of CD68‐positive and CD163‐positive macrophages in fibrosis and non‐fibrosis cases. (A and B) CD68‐positive cells were predominantly localized at the interface between fibrotic areas and lymphoid follicles (*) (CD68, 40×). (C and D) CD163‐positive cells were predominantly localized at the interface between fibrotic areas and lymphoid follicles (*) (CD163, 40×).

### 3.4 Comparison of IgG4‐ROD With vs. Without Fibrosis

We classified the IgG4‐ROD cases into two groups: non‐fibrosis (*n* = 51; fibrosis ≤ 10% of the total tissue area) and fibrosis (*n* = 49; fibrosis > 10% of the total tissue area). Patterns 1–2 corresponded to the non‐fibrosis group and Patterns 3–4 to the fibrosis group. Next, we compared these two groups with regard to clinicopathological (Table 2) and immunohistochemical (Table 3) characteristics. Compared to the non‐fibrosis group, the fibrosis group showed a significantly higher incidence of extra‐orbital involvement (*p* = 0.012). The groups also differed in the incidence of salivary gland involvement, although this difference was not significant (*p* = 0.078). Serum IgG4 levels did not significantly differ between the non‐fibrosis and fibrosis groups (*p* = 0.785), and remained similar after excluding patients with salivary gland involvement (*p* = 0.646). When the same histopathological parameters were compared between patients with and without extra‐ophthalmic organ involvement, no significant differences were identified (data not shown). Although the fibrosis group showed significantly higher sIL‐2R levels (*p* = 0.038), the two groups did not significantly differ in the proportion of patients with sIL‐2R values exceeding the reference range. Histological analysis revealed that storiform fibrosis was significantly more frequent in the fibrosis group than in the non‐fibrosis group (*p* = 0.0012) (Table 2).

**TABLE 2:** Comparison of clinicopathological characteristics between IgG4‐related ophthalmic disease cases with vs. without fibrosis.

	Non‐fibrosis (*n* = 51)	Fibrosis (*n* = 49)	*p*‐value
Demographics			
Sex, male/female	27/24	24/25	0.86
Age, years	57 (28–81)	58 (33–88)	0.62
Clinical findings			
Involvement other sites	30% (14/46)	57% (27/47)	0.012
Salivary gland	24% (11/46)	43% (20/47)	0.078
Lymph node	8.7% (4/46)	15% (7/47)	0.52
Pancreas	4.3% (2/46)	8.5% (4/47)	0.67
Retroperitoneum	4.3% (2/46)	6.4% (3/47)	1.0
Kidney	2.2% (1/46)	4.3% (2/47)	1.0
Pituitary gland	0% (0/46)	2.1% (1/47)	1.0
Lung	0% (0/46)	2.1% (1/47)	1.0
Serum IgG4, mg/dL	446 (65.5–3700)	480 (130–3600)	0.785
Serum IgG4 > normal	96% (49/51)	98% (48/49)	1.0
sIL‐2R, U/mL	530 (202–1530)	637 (121–2481)	0.038
sIL‐2R > normal	57% (25/44)	68% (28/41)	0.726
Histologic features			
Storiform fibrosis	12% (6/51)	41% (20/49)	0.0012
Obliterative phlebitis	0% (0/51)	2.0% (1/49)	0.49
Eosinophil infiltration	31% (16/51)	39% (19/49)	0.53
Germinal center hyperplasia	63% (32/51)	76% (37/49)	0.20
PTGC‐like change	31% (16/51)	37% (18/49)	0.67

*Note:* Data are presented as percentage (proportion) or median (range). The reference cutoff for serum IgG4 was 135 mg/dL. Reference ranges for sIL‐2R were institution‐specific.

Abbreviations: IgG4, immunoglobulin G4; PTGC, progressive transformation of germinal centers; sIL‐2R, soluble interleukin‐2 receptor.

**TABLE 3:** Comparison of immunohistochemical characteristics between IgG4‐related ophthalmic disease cases with vs. without fibrosis.

	Non‐fibrosis (*n* = 51)	Fibrosis (*n* = 49)	*p*‐value
IgG4‐positive plasma cells, cells/HPF	217 (43–562)	230 (57–602)	0.566
IgG4/IgG‐positive cell ratio	0.88 (0.37–1.0)	0.82 (0.30–1.0)	0.761
IgG4‐positive cells in germinal centers	35% (18/51)	49% (24/49)	0.462
FOXP3‐positive cells, cells/HPF	86 (7–356)	76 (10–230)	0.372
EBER‐positive cells in background	41% (21/51)	45% (22/49)	0.667
CD68‐positive cells, cells/HPF	117 (25.3–282)	175 (54.3–501)	< 0.001
CD163‐positive cells, cells/HPF	103 (22.7–297)	127 (71–412)	< 0.001

*Note:* Data are presented as percentage (proportion) or as median (range).

Abbreviation: HPF indicates high‐power field.

Immunohistochemical analysis showed that the fibrosis and non‐fibrosis groups did not significantly differ in the number of IgG4‐positive cells or the IgG4/IgG ratio. Similarly, the groups did not significantly differ in the number of FOXP3‐positive cells. The proportion of patients with EBER‐positive cells in the background did not significantly differ between the two groups. On the other hand, compared to the non‐fibrosis group, the fibrosis group showed significantly higher numbers of CD68‐positive cells (*p* < 0.001) and CD163‐positive cells (*p* < 0.001) (Table 3).

### 3.5 RNA ISH of *TGFB1*

Given the established role of TGF‐β as a critical mediator of fibrosis, we quantitatively analyzed *TGFB1* expression in 38 cases of IgG4‐ROD from Aichi Medical University Hospital, comprising 19 non‐fibrosis and 19 fibrosis cases (Figure 4A). For each case, we selected the region showing the highest *TGFB1* expression for intergroup comparison (Figure 4B). This analysis revealed a trend toward higher *TGFB1* expression in the non‐fibrotic group, although the difference was not statistically significant (*p* = 0.121).

**FIGURE 4:**
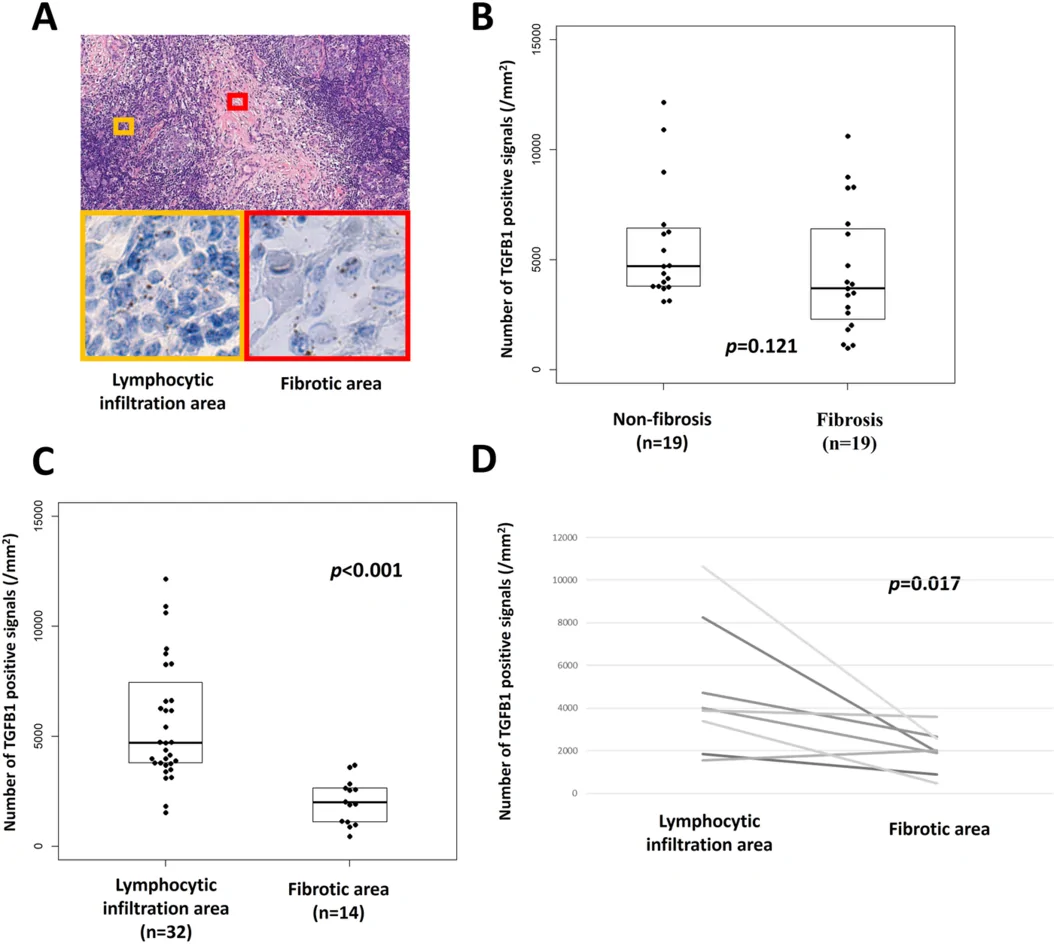
Quantitative analysis of TGFB1 expression in IgG4‐related ophthalmic disease (IgG4‐ROD) tissue. (A) Representative histological image of a specimen containing both a lymphocytic infiltration area (yellow square, enlarged below left) and a fibrotic area (red square, enlarged below right), which was stained with hematoxylin and eosin (top) and subjected to RNA in situ hybridization for *TGFB1* (bottom). Brown punctate signals in the lower panels indicate *TGFB1* mRNA expression. (B) *TGFB1* expression in the regions with the highest signal intensity was compared between the non‐fibrosis (*n* = 19) and fibrosis (*n* = 19) groups. The non‐fibrosis group showed a trend toward higher expression, although the difference was not statistically significant (*p* = 0.121). (C) Box plot comparing the number of *TGFB1*‐positive signals per mm^2^ between lymphocytic infiltration areas (*n* = 32) and fibrotic areas (*n* = 14). *TGFB1* expression was significantly higher in lymphocytic areas (*p* < 0.001). (D) Paired analysis of *TGFB1* expression in lymphocytic infiltration areas vs. fibrotic areas identified within the same specimen (*n* = 8). Intra‐case comparison revealed significantly higher *TGFB1* expression in lymphocytic areas compared to fibrotic areas (*p* = 0.017).

To further elucidate the histological context of *TGFB1* expression, entire tissue specimens were morphologically assessed and classified into lymphocytic infiltration areas and fibrotic areas. Analysis of the 38 cases revealed that *TGFB1* expression was quantifiable in the fibrotic areas of 14 cases, and in the lymphocytic infiltration areas of 32 cases. Comparative analysis demonstrated significantly higher *TGFB1* expression in the lymphocytic infiltration areas compared to the fibrotic areas (*p* < 0.001) (Figure 4C). The tissue samples from eight cases included both lymphocytic infiltration areas and fibrotic areas within the same specimen. Intra‐case analysis of these specimens revealed significantly elevated *TGFB1* expression in the lymphocytic areas compared to the fibrotic areas (*p* = 0.017) (Figure 4D).

## 4 Discussion

The comprehensive diagnostic criteria for IgG4‐RD include three pathological findings: (1) dense lymphocyte and plasma cell infiltration with fibrosis; (2) a ratio of IgG4^+^ plasma cells to IgG^+^ cells that is > 40%, and > 10 IgG4‐positive plasma cells per high‐power field (HPF); and (3) typical tissue fibrosis, particularly storiform fibrosis, or obliterative phlebitis [2]. In contrast, as shown in Supporting Information Table S1, the revised 2023 diagnostic criteria for IgG4‐ROD do not consider fibrosis and obliterative phlebitis to be key findings required for diagnosis [17]. This change was made because previous studies highlight that a substantial proportion of patients with IgG4‐ROD lack or exhibit only slight fibrosis, and describe obliterative phlebitis in only 0%–9% of cases [4, 5, 12]. In our cohort, 51 cases were classified into the non‐fibrosis group, defined as showing ≤ 10% fibrosis relative to total tissue area. Additionally, obliterative phlebitis was identified in only one case. These findings confirm that the presence of fibrosis is not required for IgG4‐ROD diagnosis and that obliterative phlebitis is rare.

Takahira et al. analyzed 38 cases that met the diagnostic criteria for IgG4‐ROD, and found that five cases failed to meet the comprehensive diagnostic criteria due to the absence of fibrosis in biopsy specimens [17]. In our cohort, 97 cases fulfilled the diagnostic criteria for definite IgG4‐ROD, whereas three cases were categorized as probable diagnoses because the serum IgG4 levels were below the reference range. On the other hand, when using the comprehensive diagnostic criteria, 28 cases would be classified as probable diagnoses due to the absence of fibrosis or low serum IgG4 levels. Explanatory Note 1 of the comprehensive diagnostic criteria states that patients categorized as having possible or probable IgG4‐RD according to the comprehensive diagnostic criteria, but who fulfill the organ‐specific criteria for IgG4‐RD, should be considered as having a definite diagnosis of IgG4‐RD. Therefore, the 25 cases that would be classified as probable under the comprehensive diagnostic criteria, due to the absence of fibrosis, may also be considered definite IgG4‐ROD. These findings further validate the diagnostic utility of the revised 2023 diagnostic criteria for IgG4‐ROD in our cohort.

We classified the analyzed IgG4‐ROD cases into four histological patterns (Patterns 1–4). Pattern 1 showed only abundant infiltration of plasma cells and lymphocytes, without lymphoid follicle formation. Pattern 2 was characterized by lymphoid follicular hyperplasia, with absent or mild fibrosis. One challenge in the pathological diagnosis of IgG4‐ROD is that Patterns 1 and 2 may not be correctly recognized as IgG4‐ROD, because many pathologists mistakenly assume that IgG4‐related disease invariably involves fibrosis. The fact that obliterative phlebitis is only rarely observed in IgG4‐ROD may further contribute to the diagnostic difficulty. Accurate diagnosis of IgG4‐ROD requires recognition that a considerable proportion of cases do not exhibit fibrosis or obliterative phlebitis.

From Pattern 1 to Pattern 4, the degree of fibrosis and sclerosis progressively increases. We hypothesize that fibrosis and sclerosis become more pronounced over time, with lesion progression. Accordingly, the histopathological features of IgG4‐ROD appear to evolve from Pattern 1 to Pattern 4, with Pattern 1 representing early‐stage lesions and Pattern 4 representing late‐stage lesions. Cheuk et al. described cases of IgG4‐related dacryoadenitis, including one case that showed morphological evolution over sequential biopsies—from predominantly florid reactive lymphoid follicular hyperplasia to a markedly atrophic gland with dense sclerosis [12]. Similarly, Ferry et al. described a case in which three sequential biopsies were performed, with the third specimen showing more pronounced fibrosis, compared to the first and second samples [5]. These observations may provide evidence supporting our hypothesis. Furthermore, in our cohort, the fibrosis group showed a significantly higher incidence of extra‐orbital involvement and higher sIL‐2R levels than the non‐fibrosis group. These findings suggest that patients in the fibrosis group underwent biopsy at a more advanced stage of the disease.

Within our cohort, EBER‐positive lymphocytes were detected in 43% (43/100) of the IgG4‐ROD cases. Among these 43 cases, 18 exhibited ≥ 5 EBV‐positive cells in at least one high‐power field, thereby meeting the criteria for EBV‐associated reactive lymphoid hyperplasia [20]. Takeuchi et al. reported increased numbers of EBER‐positive lymphocytes in 58% (18/31) of IgG4‐related lymphadenopathy cases, and 21% (5/24) of extranodal IgG4‐related disease cases. Notably, their extranodal cohort included nine cases of IgG4‐ROD, and 33% (3/9) of these cases showed increased numbers of EBER‐positive lymphocytes [21]. Taken together, these findings suggest that a subset of IgG4‐ROD cases is characterized by a significant increase of EBER‐positive cells. We speculate that IgG4‐ROD‐associated immune dysregulation may promote EBV reactivation, thereby leading to increased numbers of EBER‐positive cells [22, 23]. It remains unclear whether EBV contributes to IgG4‐ROD development. However, the proportion of patients with EBER‐positive cells did not significantly differ between the non‐fibrosis and fibrosis groups in our cohort, suggesting that EBV is unlikely to contribute to fibrosis, at least in this context.

Our analyses further revealed that the numbers of CD68‐positive and CD163‐positive cells were significantly higher in the fibrosis group compared to the non‐fibrosis group. The increased numbers of CD68‐positive and CD163‐positive macrophages in fibrotic cases may reflect enhanced monocyte recruitment during the fibrotic process. Previous studies have shown that CD4‐positive cytotoxic T cells (CTLs), granulysin‐positive CD8‐positive CTLs, and γδT cells are increased in IgG4‐RD lesions [24, 25]. These cells may induce tissue injury and create an inflammatory and profibrotic microenvironment that may promote monocyte recruitment and subsequent macrophage accumulation. We also found that these CD68‐positive and CD163‐positive cells were predominantly localized at the interface between fibrotic areas and lymphoid follicles. Chinju et al. previously reported that CD163‐positive M2 macrophages contribute to fibrosis in IgG4‐related sialadenitis, by enhancing the production of fibrotic cytokines—including TGF‐β—via activation of the TLR‐7/IRAK4/NF‐κB signaling pathway [15]. Taken together, we consider that CD163‐positive M2 macrophages may also contribute to fibrosis in IgG4‐ROD. In particular, we suggest that M2 macrophages localized within the interface between fibrotic areas and lymphoid follicles may be associated with the fibrotic process. However, because *TGFB1*‐positive cells were not characterized using dual assays with lineage‐specific markers, the present analysis could not determine whether these macrophages were a cellular source of *TGFB1*.

TGF‐β is a well‐established mediator of fibrosis, and we therefore expected *TGFB1* expression to be higher in the fibrosis group; however, *TGFB1* expression was not significantly higher in the fibrosis group than in the non‐fibrosis group [13]. Assuming that fibrosis progresses over time, these findings suggest that *TGFB1* may be produced during an early stage of the lesion. In contrast, intra‐case analysis demonstrated significantly higher *TGFB1* expression in lymphocyte‐rich areas than in fibrotic areas. Our results suggest that, in early lesions before fibrosis is fully established, TGF‐β produced within lymphocyte‐rich areas may contribute to the development of fibrosis, although the *TGFB1*‐positive cells were not specifically characterized with lineage‐specific markers in the present study. The potential contribution of lymphocytes is also supported by previous studies demonstrating TGF‐β1‐producing CD4‐positive T cells in IgG4‐RD lesions [24]. TGF‐β1 production by other inflammatory cells, including mast cells and macrophages, has also been reported in IgG4‐RD [26]. Taken together with these previous studies, our findings suggest that inflammatory cells within lymphocyte‐rich areas, including lymphocytes, may represent an important source of *TGFB1*.

In conclusion, our findings confirm that fibrosis is not essential for IgG4‐ROD diagnosis, and that obliterative phlebitis is only infrequently observed. Furthermore, we validated the diagnostic utility of the revised 2023 criteria for IgG4‐ROD in our cohort. We demonstrated that IgG4‐ROD can be categorized into four distinct histological patterns (Patterns 1–4) that exhibit a progressive increase in fibrosis and sclerosis from Pattern 1 to Pattern 4. It appears that fibrosis and sclerosis become more pronounced as lesions progress over time. Furthermore, our present results suggest that accumulation of CD163‐positive macrophages, particularly at the interface between fibrotic areas and lymphoid follicles, is associated with the fibrotic process in IgG4‐ROD.

## Author Contributions

Natsuki Taniguchi drafted the manuscript. Akira Satou drafted and revised the manuscript. Taishi Takahara analyzed the data and drafted and revised the manuscript. Yasuhiro Takahashi, Toshinobu Kubota, and Akari Iwakoshi contributed cases and revised the manuscript. Yasuharu Sato and Toyonori Tsuzuki revised and finalized the manuscript. All authors discussed the results and contributed to the final manuscript.

## Ethics Statement

This study was approved by the institutional review boards of Aichi Medical University Hospital (approval no. 2021‐066) and National Hospital Organization Nagoya Medical Center (approval no. 2022‐012) and was conducted in accordance with the Declaration of Helsinki. Informed consent was obtained using an opt‐out approach.

## Consent

The authors have nothing to report.

## Conflicts of Interest

The authors declare no conflicts of interest.

## Supporting information


**Figure S1:** Flow diagram of patient selection. A total of 106 cases diagnosed with or suspected of having IgG4‐related ophthalmic disease (IgG4‐ROD) were identified. Five cases showing light‐chain restriction were reclassified as MALT lymphoma and excluded, and one case without an available tissue block were excluded. Among the remaining 100 cases, 97 were classified as definite and 3 as probable IgG4‐ROD according to the 2023 revised diagnostic criteria.

**Figure S2:** Other histologic features of IgG4‐related ophthalmic disease. A, Storiform fibrosis (arrowhead) was identified in 26 cases (H&E, 400×). B, Obliterative phlebitis was observed in only one case (Victoria Blue, 200×). C, Eosinophil infiltration was present in 31 cases (H&E, 400×). D, Progressive transformation of germinal centers (PTGC)‐like changes were identified in 34 cases (H&E, 100×).

**Table S1:** The 2023 revised diagnostic criteria for IgG4‐related ophthalmic disease.

## Data Availability

The data that support the findings of this study are available from the corresponding author upon reasonable request.
