# Supplementary material for: Pathological Evolution of Fibrosis in IgG4‐Related Ophthalmic Disease: A Retrospective Study of 100 Cases
Source: Pathol Int. 2026 Oct 8;76(10):e70193. doi: 10.1111/pin.70193 (PMC13647486; doi:10.1111/pin.70193)
Supplement: Supplementary file 1 [file PIN-76-0-s001.docx]

Supplemental Table 1 The 2023 revised diagnostic criteria for IgG4-related ophthalmic disease

(1) Imaging studies show enlargement of the lacrimal gland, trigeminal nerve, or extraocular muscle as well as masses, enlargement, or hypertrophic lesions in various ophthalmic tissues.
(2) Histopathologic examination shows marked lymphocyte and plasmacyte infiltration, and sometimes fibrosis. A germinal center is frequently observed. IgG4⁺ plasmacytes are found and satisfy the following criteria:

 - Ratio of IgG4⁺ cells to IgG⁺ cells of 40% or above,

 - Or more than 50 IgG4⁺ cells per high-power field (×400).

(3) Blood test shows elevated serum IgG4 (>135 mg/dL).

Diagnosis is classified as:

Definitive: when (1), (2), and (3) are satisfied

Probable: when (1) and (2) are satisfied

Possible: when (1) and (3) are satisfied

Differential diagnosis of IgG4-related ophthalmic disease:

Sjögren syndrome, Lymphoma, Sarcoidosis, Granulomatosis with polyangiitis (Wegener granulomatosis), Thyroid-related orbitopathy, Idiopathic orbital inflammation, Dacryoadenitis or orbital cellulitis caused by bacteria or fungi

Attention

I) Clinicians must be vigilant for optic neuropathy, causing visual acuity and field deterioration.

Attention needs to be paid to hypertrophic pachymeningitis as the cause of optic neuropathy.

II) Lymphomas, as represented by mucosa-associated lymphoid tissue (MALT lymphoma), may contain IgG4⁺ cells; therefore, careful differentiation is necessary.
